# Treatment of experimental hyperchloremic metabolic acidosis in horses with enteral electrolyte solution containing sodium acetate

**DOI:** 10.3389/fvets.2024.1376578

**Published:** 2024-09-11

**Authors:** Lorena Chaves Monteiro, Caio Monteiro Costa, Pedro Ancelmo Nunes Ermita, Silvio José Printes Gomes Júnior, Felipe Sperandio Mattos, Fernanda Campos Mansur, Mayara Oliveira dos Santos, Samuel Rodrigues Alves, Erica Garcia Mafort, Cíntia Fernandes Fidélis, Marcel Ferreira Bastos Avanza, Raffaella Bertoni Cavalvanti Teixeira, Rinaldo Batista Viana, José Dantas Ribeiro Filho

**Affiliations:** ^1^Laborary of Research in Veterinary Internal Medicine, Veterinary Department, Universidade Federal de Viçosa, Viçosa, Brazil; ^2^Universidade Federal do Sul e Sudeste do Pará, Xinguara, Brazil; ^3^Institute of Animal Health and Production, Universidade Federal Rural da Amazônia, Belém, Brazil

**Keywords:** fluid therapy, aciduria, alkalinizing, electrolyte solutions, equine

## Abstract

**Introduction:**

In adult horses, the development and evaluation of enteral electrolyte solutions containing sodium acetate for correcting hyperchloremic metabolic acidosis are still lacking, although these electrolyte and acid–base imbalances are commonly observed. The objective of this study was to evaluate the alkalinizing effect of two enteral electrolyte solutions containing different concentrations of acetate, administered via nasogastric tube in continuous flow, in adult horses with experimental hyperchloremic metabolic acidosis.

**Methods:**

Six mares aged between 3 and 10 years were used in a 6×2 crossover design, with each animal receiving both treatments. The horses were subjected to a protocol to induce hyperchloremic metabolic acidosis. They then received one of two treatments: HighAcetate (81.4 mmol/L) and LowAcetate (22.7 mmol/L) at an infusion rate of 15 mL/kg/h for 12 h. Plasma, serum, and urinary biochemical assessments; hematocrit; urinary volume, pH, and specific gravity; and blood gas analysis were measured at the following time points: T-12 h (beginning of the 12-h fast), T0h (end of fasting and beginning of the acidosis induction phase), every 2 h during the hyperchloremic metabolic acidosis induction phase (T_i_2h and T_i_4h), every 2 h during the 12-h enteral hydration phase (T_t_2h, T_t_4h, Tt6h, T_t_8h, T_t_10h, and T_t_12h), with one sample taken at T24h (24 h after the start of acidosis induction) and another at T36h (36 h after the start of acidosis induction). Data were analyzed using descriptive statistics and analysis of variance based on a factorial design of repeated measures, with Tukey’s *post-hoc* test or the Kruskal-Wallis test with Dunn’s *post-hoc* test for non-parametric tests.

**Results:**

At the end of the induction phase, the animals developed moderate to severe hyperchloremic metabolic acidosis. The HighAcetate solution effectively corrected electrolyte and acid–base imbalances before the end of the treatment phase (Tt12h), while the LowAcetate solution was not effective in correcting those changes.

**Conclusion:**

The HighAcetate (81.4 mmol/L) solution is deemed an effective and safe alternative for the treatment of hyperchloremic metabolic acidosis in horses.

## Introduction

Hydroelectrolyte and acid–base imbalances are common in several diseases and syndromes affecting horses. However, diseases of the gastrointestinal tract are particularly important due to their impact on the animals’ homeostasis. These diseases result in the sequestration of large amounts of fluid and electrolytes, leading to acid–base disturbances (1). Metabolic acidosis is the major acid–base imbalance observed in horses with diarrhea (2, 3), colic (4), chronic kidney disease (5), after exercise (6), and in sick foals (7).

Correction of hyperchloremic metabolic acidosis is typically achieved through the administration of sodium bicarbonate intravenously or through enteral electrolyte solutions containing an alkalinizing substance administered via the nasogastric route. Currently, it is recommended to use sodium acetate in enteral electrolyte solutions, as sodium bicarbonate can interfere with the pH of the gastrointestinal tract (8–10). This interference does not occur with the use of sodium acetate (9), as its alkalizing action occurs during hepatic and muscular metabolism (11, 12). In foals, Monteiro et al. (13, 14) observed an increase in alkaline reserve following the administration of enteral electrolyte solutions containing sodium acetate. This suggests that sodium acetate is a viable alternative to sodium bicarbonate as a component of alkalinizing enteral electrolyte solutions for correcting hyperchloremic metabolic acidosis.

The absence of controlled trials evaluating the efficacy of sodium acetate in enteral electrolyte solutions for correcting hyperchloremic metabolic acidosis in adult horses, despite the common occurrence of this imbalance in the species, highlights the importance of studies in this area. The present research was designed to evaluate the efficacy of two enteral electrolyte solutions containing different concentrations of acetate, administered in continuous flow via a nasogastric tube in horses with experimentally induced hyperchloremic metabolic acidosis. Our hypothesis is that an enteral electrolyte solution containing 8 grams of sodium acetate per liter is effective in correcting experimental hyperchloremic metabolic acidosis.

## Materials and methods

### Ethics and experimental designing

This study adhered to the Standards of Conduct for Animal Use and Animal Experimentation set by the Animal Use Ethics Committee of the Federal University of Viçosa, under protocol number 20/2020.

This work was a controlled clinical trial in a cross-over design (6 × 2). Six healthy crossbred mares, aged between 3 and 10 years, not pregnant or lactating, with a good body condition score and an average weight of 344 ± 20 kg, were included in the study. Their health status was confirmed based on normal physical examinations, complete blood cell counts, and chemistry profiles. During the adaptation period, the animals were managed semi-extensively, grazing freely, and receiving chopped elephant grass (*Pennisetum purpureum*), concentrate (1% of their body weight, divided into two daily portions), mineral supplements, and water *ad libitum*.

The experimental protocol consisted of an induction phase of hyperchloremic metabolic acidosis (HyMeAcid), immediately followed by the treatment phase using continuous flow enteral fluid therapy (_CF_EFTh). Prior to the induction of HyMeAcid and the administration _CF_EFTh, the animals were subjected to 12 h of food deprivation while kept in individual stalls with rubber mats.

To minimize handling stress before the start of the induction of HyMeAcid phase, venous access was established in the jugular vein (right side at the first week of the experiment and in the left jugular vein the following week) with a 14G intravenous catheter, nasogastric intubation was performed, and the bladder was catheterized with a Foley catheter connected to a urinary collection system after wrapping the tail and disinfecting the perineal region with 2% chlorhexidine. To start the induction of HyMeAcid phase, the catheter was connected to a macrodrop line and a one-liter bag of acidifying solution.

### Induction of hyperchloremic metabolic acidosis

The induction of HyMeAcid was achieved by the intravenous administration of a sterile crystalloid solution (indSOL), containing 152.5 mmol of sodium and 252.5 mmol of chloride per 1,000 mL of solution. The indSOL was adapted from the protocol described by Romão et al. (2017). The total volume of indSOL administered intravenously to each animal was equivalent to 7.5% of its body weight over a four-hour period. The infusion rate was 18.75 mL/kg/h.

### Treatment of hyperchloremic metabolic acidosis

After the induction of HyMeAcid the animals were treated with one of two enteral electrolyte solutions, HighAcetate and LowAcetate (Tables 1, 2). The administration of enteral electrolyte solutions began immediately after the HyMeAcid induction phase. The solutions were administered in a continuous flow via the nasogastric route. The average total volume administered to each animal was 61.92 liters, infused at a rate of 15 mL/kg/h for 12 h.

**Table 1:** Composition of HighAcetate and LowAcetate treatments in g/L of enteral electrolyte solution.

Substance	HighAcetate	LowAcetate
NaCl	3 g/L	3 g/L
KCl	0.5 g/L	0.5 g/L
MgCl_2_ * Hexahydrate	0.3 g/L	0.3 g/L
Calcium Acetate Monohydrate	2 g/L	2 g/L
Sodium Acetate Trihydrate	8 g/L	0
Dextrose	10 g/L	10 g/L

**Table 2:** Measured osmolarity (mOsm/L), measured concentrations of electrolytes and glucose (mmol/L), and calculated concentrations of acetate (mmol/L) in the HighAcetate and LowAcetate treatments.

Substance	HighAcetate	LowAcetate
Na^+^	110	67
K^+^	6.7	6.7
Cl^−^	61	61
Ca^2+^	11.4	11.4
Mg^2+^	1.5	1.5
Glucose	55.5	55.5
Acetate	81.4	22.7
Osmolarity	290	196

The animals were kept on food and water deprivation until the end of the hydration phase (T_T_12h), after which they were turned out to pasture and fed free choice *Tifton 85* hay and water *ad libitum* until T36h. Each animal received both solutions with a seven-day interval between treatments. This interval was determined based on results from previously conducted pilot studies to ensure no overlap in the effects of the treatments.

### Blood and urine samples were collected at the following time points

T-12 h: at the start of the food deprivation phase; T_i_0h: at the end of the water and food deprivation phase and the beginning of the HyMeAcid induction phase; T_i_2h: 2 h after the start of the HyMeAcid induction phase; T_i_4h: 4 h after the start and at the end of the HyMeAcid induction phase; T_T_2h, T_T_4h, T_T_6h, T_T_8h, T_T_10h, and T_T_12h: during the administration of enteral electrolyte solutions (treatment phase); T24h and T36h: 24 and 36 h after the end of the HyMeAcid induction phase.

#### Collection of biological samples and laboratory evaluation

Venous blood samples at T-12 h, T24h, and T36h were collected through jugular venipuncture using a vacuum system. In the other moments of evaluation, the samples were obtained directly from the venous catheter in the external jugular vein. After the end of the induction of hyperchloremic metabolic acidosis, the catheter was sealed with a luer lock adapter and filled with 2 mL of saline solution containing sodium heparin (100 IU/mL). Before each collection, 5 mL of blood was aspirated directly from the catheter and discarded along with the syringe. At the end of each collection, the catheter was filled with 2 mL of the heparin solution.

Blood samples were placed in tubes containing a clot activator and kept in a water bath at 37°C for 40 min to form the clot. Serum separation was performed by centrifugation. Blood samples were also collected in tubes containing sodium fluoride and EDTA and centrifuged immediately after collection to obtain plasma. The serum and plasma samples was then stored at −20°C until analysis.

Serum concentrations of total calcium (Arsenazo III, InVitro Diagnóstica, Minas Gerais, Brazil), magnesium (Xylidyl Blue, InVitro Diagnóstica, Minas Gerais, Brazil), urea (GLDH, InVitro Diagnóstica, Minas Gerais, Brazil), and creatinine (Jaffé, InVitro Diagnóstica, Minas Gerais, Brazil), were measured. Plasma concentrations of glucose (Colorimetric Enzymatic GOD, InVitro Diagnóstica, Minas Gerais, Brazil) and lactate (Enzymatic LDH, InVitro Diagnóstica, Minas Gerais, Brazil) were also measured. The analyses were performed using an automatic biochemistry device (HumaStar 300 Automated Chemistry Analyzer, Human Diagnostics, Wiesbaden, Germany).

#### Hemogasometry, electrolytes, anion gap, SID and Atot calculation

Blood samples for blood gas analysis (pH, pCO₂, HCO₃^−^, and BE) and electrolyte measurement (Na^+^, K^+^, Cl^−^, iCa^2+^) were obtained in syringes containing lithium heparin. The analyses were performed immediately after venous blood sample collection (ABL 80 Flex—Radiometer—Copenhagen, Denmark). The blood values of pH, pCO_2_, blood bicarbonate (HCO_3_^−^), base excess (BE), anion gap, sodium, potassium, chloride and ion calcium were determined. The anion gap, strong ion difference (SID), and plasma concentration of weak acids (Atot) were calculated using the following equations (15, 16):

$$\mathrm{Anion}\;\mathrm{gap}\;\left(\mathrm{mmol}/L\right)=\left(\left[Na^{+}\right]+\left[K^{+}\right]\right)-\left(\left[Cl^{-}\right]+\left[HCO_{3}^{-}\right]\right)$$

$$SID\;\left(\mathrm{mmol}/L\right)=\left(\left[Na^{+}\right]+\left[K^{+}\right]\right)-\left[{Cl}^{-}\right]$$

$$\mathrm{Atot}\;\left(\mathrm{mmol}/L\right)=\left(2.2\times PPT^{*}\right)$$

*PPT is total plasma protein in g/dL.

#### Urinary biochemical analysis

For urinary assessment, at each time point, a 50 mL aliquot of urine was obtained directly from the bladder through a Foley catheter. Immediately after each urine collection, the pH was measured, an aliquot was centrifuged, and the supernatant was stored at −20°C for subsequent biochemical analysis.

Urinary sodium and potassium concentrations were measured using a flame photometer (B462 - Micronal, São Paulo, Brazil). Urinary chloride (Mercuric Thiocyanate, InVitro Diagnóstica, Minas Gerais, Brazil), calcium (Arsenazo III, InVitro Diagnóstica, Minas Gerais, Brazil) and magnesium (Xylidyl Blue, InVitro Diagnóstica, Minas Gerais, Brazil) concentrations were measured on an automated clinical chemistry device (HumaStar 300 Automated Chemistry Analyzer, Human Diagnostics, Wiesbaden, Germany).

To determine the urinary flow (mL/min), the volume of urine produced by each animal at each time point (mL) was measured and divided by the duration of each period (120 min). At times T-12 h, T_i_0h, T24h, and T36h, urinary flow (mL/min) was calculated from the volume of urine produced over 30 min.

Urinary electrolyte excretion (C = mmol/min) was calculated using the following formula (13):

$$C=U_{x}\times V$$

*Where U_x_ is the concentration of the substance in the urine (mmol/mL) and V is the urinary flow rate.

#### Graduation of acid–base disorders

The intensity of the acid–base imbalances was classified according to the scale proposed by Ribeiro Filho, which was developed based on his clinical experience, as outlined in Table 3.

**Table 3:** Classification of acid–base imbalances in adult horses.

Intensity	Blood pH (7.38–7.44)	Base excess (0–5 mmol/L)
Slight metabolic acidosis	7.30–7.20	Up to −6
Moderate metabolic acidosis	7.19–7.10	−7 to − 15
Severe metabolic acidosis	≤7.09	≤ −16
Slight metabolic alkalosis	7.46–7.55	6 to 12
Moderate metabolic alkalosis	7.56–7.65	13 to 19
Intense metabolic alkalosis	≥ 7.66	≥ 20

### Statistical data analysis

The data were subjected to descriptive analysis to obtain the mean and standard deviation. The normality of data distribution and the sphericity of variances were evaluated using the Shapiro–Wilk and Mauchly tests, respectively. The main effects of time, treatments, and the time*treatment interaction were evaluated by ANOVA based on a repeated measures factorial scheme. When necessary, the Tukey *post-hoc* test was used. For the qualitative variables and other variables that did not meet the ANOVA assumptions, the effect of time was evaluated using the non-parametric Kruskal-Wallis test followed by Dunn’s *post-hoc* test, and the treatment effect at each time point was evaluated using Wilcoxon’s test. All analyses were performed using SPSS version 25 (IBM, SPSS, Chicago), and significance was considered when *p* < 0.05.

#### Results

The results of the evaluated variables are described in Tables 4–10. The protocol for inducing HyMeAcid caused moderate metabolic acidosis in the animals with no adverse effects. From T_i_2h until the end of the induction phase (T_i_4h), the animals exhibited mild drowsiness. However, during the treatment phase, the animals remained alert and exhibited no behavioral changes.

**Table 4:** Biochemical parameters [mean and standard deviations of lactate, urea, and creatinine; median and interquartile range of glucose] of horses with experimentally induced hyperchloremic metabolic acidosis, treated with two enteral electrolyte solutions HighAcetate (HIGH) and LowAcetate (LOW).

	Fasting	Induction of acidosis			Treatment with enteral electrolyte solutions						Clinical observation	
Groups	T − 12 h	T_i_0h	T_i_2h	T_i_4h	T_T_2h	T_T_4h	T_T_6h	T_T_8h	T_T_10h	T_T_12h	T24h	T36h
*Glucose (mg/dL)												
HIGH	99 (32)^bcd^	95 (11) 7^cd^	85 (19)^d^	93 (15)^cd^	147 (25)^a^	155 (61)^a^	140 (45)^a^	122 (46)^ab^	122 (50)^ac^	137 (52)^a^	105 (51)^bcd^	96 (20)^bcd^
LOW	93 (15)^b^	91 (17)^b^	88 (6)^b^	94 (12)^b^	153 (45)^a^	168 (14)^a^	157 (17)^a^	157 (24)^ac^	156 (18)^a^	150 (22)^ac^	103 (5)^bc^	99 (8)^b^
Lactate (mmol/L)												
HIGH	1.4 ± 0.3^abc^	1.3 ± 0.1^bc^	1.2 ± 0.1^bc^	1.1 ± 0.1^c^	1.3 ± 0.2^abc^	1.4 ± 0.1^abc^	1.5 ± 0.1^a^	1.6 ± 0.2^a^	1.6 ± 0.2^a^	1.5 ± 0.2^abc^	1.3 ± 0.2^abcd^	1.5 ± 0.2^ab^
LOW	1.3 ± 0.4^abc^	1.4 ± 0.1^abc^	1.2 ± 0.1^bc^	1.1 ± 0.1^c^	1.3 ± 0.1^abc^	1.4 ± 0.1^abc^	1.4 ± 0.1^abc^	1.5 ± 0.1^ab^	1.5 ± 0.1^ab^	1.4 ± 0.1^abc^	1.3 ± 0.1^abc^	1.4 ± 0.1^abc^
Urea (mg/dL)												
HIGH	23 ± 6^ab^	23 ± 7^ab^	25 ± 8^ab^	21 ± 6^b^	20 ± 6^b^	20 ± 8^b^	19 ± 6^b^	20 ± 5^b^	18 ± 5^b^	18 ± 6^b^	28 ± 4^ab^	26 ± 4^ab^
LOW	25 ± 6^ab^	28 ± 5^ab^	24 ± 4^ab^	23 ± 3^ab^	24 ± 6^ab^	21 ± 4^b^	21 ± 3^b^	20 ± 3^b^	19 ± 2^b^	19 ± 2^b^	32 ± 3^a^	33 ± 5^a^
Creatinine (mg/dL)												
HIGH	1.3 ± 0.4	1.4 ± 0.3	1.1 ± 0.2	1.1 ± 0.2	1.1 ± 0.2	1.1 ± 0.3	1.2 ± 0.2	1.1 ± 0.2	1.1 ± 0.2	1.2 ± 0.2	1.2 ± 0.2	1.3 ± 0.1
LOW	1.3 ± 0.3	1.3 ± 0.3	1.1 ± 0.2	1.0 ± 0.2	1.1 ± 0.2	1.1 ± 0.2	1.1 ± 0.2	1.1 ± 0.2	1.1 ± 0.2	1.0 ± 0.3	1.1 ± 0.2	1.3 ± 0.2

Factorial ANOVA of repeated measures. Averages followed by different superscript lower-case letters in the same row differ among times by Tukey test (*p* < 0.05). ^*^Nonparametric variable subjected to Kruskal-Walli’s test, means followed by different lowercase superscript letters in the same row differ between and means followed by different superscript capital letters in the same column differ between treatments by Dunn’s test (*p* < 0.05).

**Table 5:** Blood gas parameters [mean and standard deviations of blood pH, pCO_2_, blood bicarbonate (HCO_3_^−^); median and interquartile range of base excess (BE)] of horses with experimentally induced hyperchloremic metabolic acidosis, treated with two enteral electrolyte solutions HighAcetate (HIGH) and LowAcetate (LOW).

Groups	Fasting	Induction of acidosis			Treatment with enteral electrolyte solutions						Clinical observation	
	T-12 h	T_i_0h	T_i_2h	T_i_4h	T_T_2h	T_T_4h	T_T_6h	T_T_8h	T_T_10h	T_T_12h	T24h	T36h
^⁑^*pH*												
HIGH	7.4 ± .02^Aabc^	7.4 ± .01^Aabc^	7.2 ± .05^Aef^	7.2 ± .06^Af^	7.3 ± .03^Ade^	7.3 ± .03^Ad^	7.3 ± .04^Ad^	7.4 ± .05^Acd^	7.4 ± .04^Aabc^	7.4 ± .02^Aab^	7.4 ± .02^Aa^	7.4 ± .02^Aab^
LOW	7.4 ± .02^Aa^	7.4 ± .02^Aa^	7.2 ± .02^Ac^	7.1 ± .03^Ad^	7.2 ± .02^Abc^	7.3 ± .02^Ab^	7.2 ± .02^Bb^	7.3 ± .03^Bb^	7.3 ± .03^Bb^	7.3 ± .03^Bb^	7.4 ± .02^Ba^	7.4 ± .03^Aa^
pCO_2_ (mmHg)												
HIGH	47 ± 5^Aa^	44 ± 2^Aabc^	40 ± 2^Acd^	36 ± 2^Ad^	40 ± 2^Acd^	40 ± 2^Acd^	41 ± 2^Abcd^	42 ± 2^Aabc^	46 ± 3^Aab^	45 ± 3^Aabc^	47 ± 3^Aa^	45 ± 2^Aabc^
LOW	45 ± 3^Aa^	45 ± 4^Aab^	38 ± 3^Acd^	35 ± 3^Ad^	38 ± 2^Acd^	38 ± 2^Acd^	40 ± 3^Aabcd^	40 ± 3^Aabcd^	39 ± 3^Bbcd^	41 ± 1^Aabcd^	41 ± 3^Aabcd^	45 ± 4^Aa^
^⁑^*HCO_3_^−^ (mmol/L)*												
HIGH	27.8 ± 1^Aab^	27.8 ± 1^Aab^	15 ± 2^Aef^	12.7 ± 2^Af^	17.4 ± 2^Ade^	18.6 ± 2^Ade^	19.8 ± 2^Acd^	22.9 ± 3^Ac^	26.7 ± 3^Ab^	29.7 ± 2^Aab^	30.8 ± 1^Aa^	29.5 ± 2^Aab^
LOW	27.8 ± 2^Aa^	26.9 ± 2^Aab^	14.4 ± 1^Ade^	11 ± 1^Ae^	15.4 ± 1^Acd^	15.9 ± 1^Acd^	16.3 ± 1^Acd^	16.4 ± 1^Bcd^	17.5 ± 2^Bcd^	18.6 ± 1^Bc^	23.5 ± 2^Bb^	25.7 ± 2^Bb^
*BE (mmol/L)												
HIGH	3 (1)^Aac^	3 (2)^Aab^	-12 (4)^Ad^	−15 (4)^Ad^	−9 (3)^Acd^	−7 (3)^Abcd^	−6 (6)^Abcd^	−2 (6)^Abc^	2 (6)^Aab^	5 (5)^Aa^	7 (2)^Aa^	5 (3)^Aa^
LOW	4 (2)^Aa^	3 (4)^Aa^	−12 (2)^Ade^	−16 (3)^Ae^	−11 (2)^Bde^	−10 (1)^Bcde^	−10 (1)^Bbcd^	−10 (3)^Bcd^	−8 (4)^Bbcd^	−7 (4)^Bac^	−1 (2)^Bab^	1 (3)^Ba^

Factorial ANOVA of repeated measures. Averages followed by different superscript lower-case letters in the same row differ among times and averages followed by different superscript upper-case letters in the same column differ among treatments by Tukey test (*p* < 0.05). ^*^Nonparametric variable subjected to Kruskal-Walli’s test, means followed by different lowercase superscript letters in the same row differ between and means followed by different superscript capital letters in the same column differ between treatments by Dunn’s test (*p* < 0.05). ^⁑^There was interaction between treatment and time.

**Table 6:** Mean values and standard deviations of the hematological parameters [strong ion difference (SID), weak acid plasma concentration (Atot), and anion gap] of horses with experimentally induced hyperchloremic metabolic acidosis, treated with two enteral electrolyte solutions HighAcetate (HIGH) and LowAcetate (LOW).

Groups	Fasting	Induction of acidosis			Treatment with enteral electrolyte solutions						Clinical observation	
	T-12 h	T_i_0h	T_i_2h	T_i_4h	T_T_2h	T_T_4h	T_T_6h	T_T_8h	T_T_10h	T_T_12h	T24h	T36h
^⁑^SID (mmol/L)												
HIGH	43 ± 3^Aabc^	44 ± 2^Aa^	37 ± 2^Ade^	35 ± 3^Ae^	38 ± 2^Acde^	39 ± 3^Acde^	39 ± 2^Abcde^	41 ± 3^Aabcd^	44 ± 3^Abc^	44 ± 1^Aabc^	44 ± 3^Aab^	44 ± 2^Aabc^
LOW	41 ± 2^Aabc^	44 ± 3^Aa^	36 ± 2^Ad^	34 ± 3^Ad^	36 ± 2^Acd^	37 ± 2^Acd^	38 ± 2^Acd^	37 ± 2^Acd^	37 ± 3^Bcd^	38 ± 3^Bbcd^	38 ± 4^Bcd^	39 ± 3^Abcd^
Atot (mmol/L)												
HIGH	15 ± 0,7^abc^	16 ± 0,8^a^	14 ± 0,5^bc^	14 ± 0,8^c^	14 ± 0,7^abc^	15 ± 0,7^abc^	16 ± 0,5^abc^	15 ± 0,5^abc^	15 ± 0,8^abc^	16 ± 0,8^ab^	15 ± 1,0^abc^	14 ± 0,5^abc^
LOW	15 ± 0,5^ab^	16 ± 0,9^a^	14 ± 0,8^b^	14 ± 0,8^b^	15 ± 0,7^ab^	15 ± 0,8^ab^	15 ± 0,9^ab^	16 ± 1,1^ab^	16 ± 1,0^a^	16 ± 0,9^a^	15 ± 1,1^ab^	15 ± 1,0^ab^
^**⁑**^Anion gap (mmol/L)												
HIGH	15 ± 2^cde^	17 ± 1^bcde^	22 ± 2^a^	23 ± 2^a^	21 ± 1^ab^	20 ± 2^ab^	20 ± 1^abc^	18 ± 2^abcd^	17 ± 1^bcde^	15 ± 2^cd^	13 ± 1^e^	14 ± 3^de^
LOW	14 ± 2^d^	17 ± 2^bcd^	22 ± 2^a^	23 ± 2^a^	21 ± 2^ab^	21 ± 2^ab^	22 ± 1^ab^	21 ± 2^ab^	20 ± 2^abc^	20 ± 2^abc^	14 ± 4^d^	13 ± 4^d^

Factorial ANOVA of repeated measures. Averages followed by different superscript lower-case letters in the same row differ among times and averages followed by different superscript upper-case letters in the same column differ among treatments by Tukey test (*p* < 0.05). ^⁑^There was interaction between treatment and time.

**Table 7:** Serum electrolytes [mean values and standard deviations of sodium, and chloride, and median and interquartile range of potassium] of horses with experimentally induced hyperchloremic metabolic acidosis, treated with two enteral electrolyte solutions HighAcetate (HIGH) and LowAcetate (LOW).

Groups	Fasting	Induction of acidosis			Treatment with enteral electrolyte solutions						Clinical observation	
	T-12 h	T_i_0h	T_i_2h	T_i_4h	T_T_2h	T_T_4h	T_T_6h	T_T_8h	T_T_10h	T_T_12h	T24h	T36h
Na^+^ (mmol/L)												
HIGH	137 ± 2^bc^	135 ± 1^c^	141 ± 1^ab^	143 ± 2^a^	139 ± 2^abc^	139 ± 2^abc^	139 ± 3^abc^	139 ± 3^abc^	139 ± 2^abc^	139 ± 3^abc^	137 ± 2^bc^	134 ± 2^c^
LOW	136 ± 2^c^	135 ± 2^c^	141 ± 2^ab^	145 ± 2^a^	139 ± 1^bc^	139 ± 1^bc^	139 ± 3^bc^	137 ± 4^bc^	136 ± 5^bc^	137 ± 3^bc^	137 ± 3^bc^	136 ± 1^bc^
^*^K^+^ (mmol/L)												
HIGH	3.8 (1.3)^Aabcd^	4.1 (0.4)^Aabc^	4 (0.4)^Aabc^	4.7 (1.3)^Aa^	4.4 (1)^Aab^	3.8 (0.3)^Abcd^	3.6 (0.3)^Bcd^	3.5 (0.3)^Bd^	3.5 (0.2)^Bd^	3.5 (0.4)^Bd^	3.4 (0.5)^Ad^	3.8 (1.1)^Abcd^
LOW	3.8 (1.2)^Ab^	4 (0.3)^Ab^	4 (0.7)^Ab^	4.9 (0.7)^Aa^	4.8 (1.2)^Aa^	4.4 (0.6)^Aab^	4.1 (0.5)^Ab^	4.3 (0.8)^Aab^	4.2 (0.6)^Aab^	4.2 (1.0)^Aab^	4 (1.7)^Ab^	4.3 (1.3)^Aab^
^⁑^Cl^−^ (mmol/L)												
HIGH	97 ± 2^Ade^	94 ± 1^Ae^	108 ± 2^Aab^	112 ± 3^Aa^	105 ± 3^Abc^	104 ± 3^Abc^	103 ± 3^Abc^	101 ± 3^Acd^	99 ± 4^Acd^	97 ± 4^Bde^	96 ± 3^Be^	94 ± 3^Be^
LOW	98 ± 2^Acd^	95 ± 1^Ad^	109 ± 1^Ab^	115 ± 1^Aa^	108 ± 1^Abc^	107 ± 1^Abc^	106 ± 1^Abc^	104 ± 2^Abc^	104 ± 2^Ac^	103 ± 2^Ac^	103 ± 1^Ac^	102 ± 2^Ac^

Factorial ANOVA of repeated measures. Averages followed by different superscript lower-case letters in the same row differ among times and averages followed by different superscript upper-case letters in the same column differ among treatments by Tukey test (*p* < 0.05). ^*^Nonparametric variable subjected to Kruskal-Walli’s test, means followed by different lowercase superscript letters in the same row differ between and means followed by different superscript capital letters in the same column differ between treatments by Dunn’s test (*p* < 0.05). ^⁑^There was an interaction between treatment and time.

**Table 8:** Serum electrolytes [median and interquartile range of ionic calcium, and mean and standard deviations total calcium, and total magnesium] of horses with experimentally induced hyperchloremic metabolic acidosis, treated with two enteral electrolyte solutions HighAcetate (HIGH) and LowAcetate(LOW).

Groups	Fasting	Induction of acidosis			Treatment with enteral electrolyte solutions						Clinical observation	
	T-12 h	T_i_0h	T_i_2h	T_i_4h	T_T_2h	T_T_4h	T_T_6h	T_T_8h	T_T_10h	T_T_12h	T24h	T36h
^*^Ca_i_^2+^ *(mmol L^−1^)*												
HIGH	1.7 (0.1)^Abc^	1.5 (0.2)^Ab^	1.6 (0.30)^Ab^	1.6 (0.3)^Abc^	2.1 (0.1)^Aa^	2.3 (0.3)^Aa^	2.3 (0.5)^Aa^	2.2 (0.6)^Aa^	2.2 (0.6)^Aa^	2.1 (0.5)^Bac^	1.6 (0.2)^Bb^	1.6 (0.2)^Ab^
LOW	1.6 (0.3)^Ac^	1.6 (0.03)^Ac^	1.7 (0.1)^Abc^	1.7 (0.2)^Abc^	2.2 (0.2)^Aab^	2.3 (0.2)^Aa^	2.5 (0.3)^Aa^	2.5 (0.5)^Aa^	2.5 (0.5)^Aa^	2.6 (0.5)^Aa^	1.8 (0.2)^Abc^	1.6 (0.1)^Abc^
^⁑^Ca_total_^2+^ (mg dL^−1^)												
HIGH	12.7 ± 1.2^bc^	12.2 ± 0.8^bc^	10.7 ± 0.9^c^	10.4 ± 1.4^c^	13.8 ± 1^ab^	14.5 ± 0.9^ab^	15.5 ± 1.3^a^	15.6 ± 1.6^a^	15.1 ± 2.2^ab^	16.0 ± 0.8^a^	12.8 ± 0.9^bc^	12.2 ± 0.3^bc^
LOW	11.3 ± 1.3^d^	12.2 ± 0.5^cd^	10.2 ± 0.6^d^	10.2 ± 0.5^d^	13.3 ± 1^abcd^	14.5 ± 0.7^abc^	15.4 ± 0.7^ab^	15.8 ± 1.1^a^	15.9 ± 1.4^a^	15.7 ± 2.1^ab^	12.4 ± 0.5^cd^	12.3 ± 0.4^cd^
^⁑^Mg^2+^ (mg dL^−1^)												
HIGH	3.2 ± 0.5^ab^	2.2 ± 0.6^abc^	1.8 ± 0.4^abc^	1.7 ± 0.3^abc^	2.0 ± 0.3^abc^	1.7 ± 0.4^abc^	1.6 ± 0.3^bc^	1.5 ± 0.3^bc^	1.4 ± 0.3^c^	1.6 ± 0.6^bc^	2.7 ± 0.5^abc^	3.3 ± 0.5^a^
LOW	3.1 ± 0.8^ab^	2.5 ± 0.5^ab^	2.1 ± 0.6^ab^	2.0 ± 0.6^ab^	2.6 ± 0.9^ab^	2.2 ± 0.6^ab^	2.5 ± 1.3^ab^	2.6 ± 1.4^ab^	2.5 ± 1.3^ab^	2.4 ± 1.2^ab^	3.3 ± 1.2^a^	3.1 ± 0.5^ab^

Factorial ANOVA of repeated measures. Averages followed by different superscript lower-case letters in the same row differ among times and averages followed by different superscript upper-case letters in the same column differ among treatments by Tukey test (*p* < 0.05). *Nonparametric variable subjected to Kruskal-Walli’s test, means followed by different lowercase superscript letters in the same row differ between and means followed by different superscript capital letters in the same column differ between treatments by Dunn’s test (*p* < 0.05). ^⁑^There was an interaction between treatment and time.

**Table 9:** Median and interquartile range of urinary biochemical profile [total calcium, total magnesium, sodium, potassium, and chloride] of horses with experimentally induced hyperchloremic metabolic acidosis, treated with two enteral electrolyte solutions HighAcetate (HIGH) and LowAcetate (LOW).

Groups	Fasting	Induction of acidosis			Treatment with enteral electrolyte solutions						Clinical observation	
	T-12 h	T_i_0h	T_i_2h	T_i_4h	T_T_2h	T_T_4h	T_T_6h	T_T_8h	T_T_10h	T_T_12h	T24h	T36h
*Ca_t_^2+^ (mg/min)												
HIGH	0,7 (1,2) ^de^	0.2 (0.6) ^e^	1.7 (1.5) ^cde^	6.7 (3.6) ^bcd^	12 (11.3)^ac^	20 (10)^ab^	22.6 (16.4) ^a^	22.8 (11.9) ^ab^	23.7 (14.6) ^ab^	22.7 (24.1)^ab^	6.8 (12)^bcd^	0.9 (1.6)^de^
LOW	0.4 (1.7)^d^	0.4 (1.0)^d^	1.4 (2.1)^cd^	7.7 (3.3)^cd^	7.9 (11.3)^bc^	21 (12.4)^ab^	23.7 (9.9)^ab^	23.5 (11)^ab^	23.9 (11.4)^ab^	33.8 (6.9)^a^	11.6 (10.5)^bc^	6.34 (15.9)^cd^
*Mg^2+^ (mg/min)												
HIGH	0.3 (0.3) ^abc^	0.08 (0.06)‘^d^	0.06 (0.07)^d^	0.2 (0.1) ^abc^	0.3 (0.3) ^ab^	0.3 (0.06)^abc^	0.3 (0.1)^a^	0.2 (0.1)^abc^	0.1 (0.1)^c^	0.2 (0.2)^abc^	0.4 (0.4)^ab^	0.2 (0.3)^abc^
LOW	0.2 (0.2)^bd^	0.05 (0.07)^d^	0.11 (0.08)^cd^	0.2 (0.1)^abc^	0.3 (0.2)^ab^	0.3 (0.2)^ab^	0.3 (0.2)^ab^	0.3 (0.1)^ab^	0.2 (0.1)^abc^	0.3 (0.2)^ab^	0.4 (0.2)^a^	0.4 (0.4)^a^
*Na^+^ *(mEq/min)*												
HIGH	0.02 (0.1)^Ac^	0.6 (0.2)^Ac^	5.0 (4.7)^Aabc^	21.2 (9.5)^Aa^	6.8 (7.7)^Aab^	4.4 (3.8)^Aabc^	4.6 (4.0)^Aabc^	4.7 (3.8)^Aabc^	5.0 (4.1)^Aabc^	7.6 (2.4)^Aab^	3.4 (4.8)^Aabc^	1.2 (1.7)^Abc^
LOW	0.05 (0.05)^Ac^	0.1 (0.3)^Ab^	6.8 (5.9)^Aab^	23.5 (7.9)^Aa^	8.1 (4.3)^Aa^	6.3 (3.6)^Aab^	4.6 (2.9)^Aabc^	5.3 (2.4)^Aabc^	5.2 (3.4) ^Aabc^	6.5 (2.3)^Aab^	1.1 (1.6)^Bbc^	0.4 (1.0)^Abc^
*K^+^ (mEq/min)												
HIGH	1.6 (1.4)^a^	0.8 (0.6)^ab^	0.6 (0.2)^b^	2.2 (0.8)^a^	2.2 (1.6)^a^	1.2 (0.6)^ab^	1.1 (0.7)^ab^	1.1 (1.1)^ab^	0.7 (0.5)^b^	0.9 (0.7)^ab^	1.7 (1.3)^a^	1.1 (2.6)^ab^
LOW	1. (0.7)^ab^	0.6 (0.7)^b^	0.6 (0.5)^b^	1.9 (0.5)^a^	2.3 (1.4)^a^	1.6 (1.1)^ab^	1.1 (0.4)^ab^	1.1 (0.2)^ab^	0.9 (0.7)^ab^	1.1 (1.0)^ab^	1.6 (1.3)^ab^	1.03 (1.0)^ab^
*Cl^−^ (mEq/min)												
HIGH	0.9 (0.7) ^Acd^	0.5 (0.2) ^Ad^	3.5 (2.5)^Abc^	13.8 (4.7)^Aa^	5.7 (4)^Aab^	5.1 (1.5)^Aab^	4.8 (2.9)^Abe^	4.5 (1.1)^Bbe^	5.2 (3.1)^Aabe^	6.1 (3.5)^Aab^	2.2 (1.1)^Acde^	0.8 (0.5)^Ad^
LOW	0.5 (0.2)^Acd^	0.4 (0.2)^Bcd^	4.2 (4.1)^Abcd^	16.0 (4.4)^Aa^	6.1 (2.4)^Aab^	6.6 (3.0)^Aab^	5.5 (1.0)^Abcd^	5.7 (1.4)^Abc^	5.7 (3.1)^Abc^	7.7 (1.1)^Aab^	1.3 (0.9)^Acd^	0.7 (0.6)^Ad^

Factorial ANOVA of repeated measures. Averages followed by different superscript lower-case letters in the same row differ among times and averages followed by different superscript upper-case letters in the same column differ among treatments by Tukey test (*p* < 0.05).

**Table 10:** Median and interquartile range values of urinary biochemical profile [urinary volume and urinary pH] of horses with experimentally induced hyperchloremic metabolic acidosis, treated with two enteral electrolyte solutions HighAcetate (HIGH) and LowAcetate (LOW).

Groups	Fasting	Induction of acidosis			Treatment with enteral electrolyte solutions						Clinical observation	
	T-12 h	T_i_0h	T_i_2h	T_i_4h	T_T_2h	T_T_4h	T_T_6h	T_T_8h	T_T_10h	T_T_12h	T24h	T36h
*Urinary Volume (mL/min)												
HIGH	9 (7)^Ac^	5 (4)^Ac^	31 (19)^Abc^	118 (47)^Aa^	50 (23)^Aab^	43 (29)^Bb^	44 (35)^Ab^	39 (14)^Bb^	52 (39)^Aab^	64 (17)^Bab^	16 (19)^Abc^	10 (9)^Ac^
LOW	5 (1)^Ae^	3 (2)^Ae^	37 (34)^Abde^	136 (45)^Aa^	55 (20)^Abcd^	72 (33)^Aab^	60 (21)^Abcd^	65 (19)^Aab^	75 (26)^Aab^	86 (20)^Aac^	7 (6)^Ade^	5 (4)^Ae^
*Urinary pH												
HIGH	8.1 (1.2)^Aa^	8.6 (0.8)^Aa^	5.2 (1.0)^Abc^	4.8 (0.7)^Acd^	4.5 (0.5)^Ad^	4.5 (0.9)^Ad^	4.5 (0.4)^Ad^	5.1 (2.8)^Abd^	6.5 (2.3)^Aab^	7.4 (1.5)^Aab^	8.4 (0.5)^Aa^	9 (1.3)^Aa^
LOW	8.7 (2.2)^Aa^	8.2 (1.7)^Aab^	4.8 (0.4)^Abcd^	4.6 (0.4)^Acde^	4.5 (0.6)^Acde^	4.4 (0.5)^Acde^	4.3 (0.5)^Ade^	4.4 (0.3)^Ae^	4.4 ± 0.3^Bcde^	4.4 (0.3)^Bcde^	4.9 (1.0)^Babc^	5.6 (1.3)^Bab^

Factorial ANOVA of repeated measures. Averages followed by different superscript lower-case letters in the same row differ among times and averages followed by different superscript upper-case letters in the same column differ among treatments by Tukey test (*p* < 0.05). *Nonparametric variable subjected to Kruskal-Walli’s test, means followed by different lowercase superscript letters in the same row differ between and means followed by different superscript capital letters in the same column differ between treatments by Dunn’s test (*p* < 0.05).

No difference between treatments were observed for the evaluated variables during the HyMeAcid induction phase (T_i_0h to T_i_4h; *p* > 0.05), except for urinary chloride excretion in T_i_0h. Animals from all treatments showed a reduction in the value of blood pH, pCO_2_, blood HCO_3_^−^, base excess, SID, Atot, and urinary pH (Tables 4, 5, 10). There was a progressive increase in the anion gap and blood concentrations of sodium, potassium, and chloride (*p* < 0.05), reaching their maximum values at T_i_4h. An increase in urinary excretion of all measured electrolytes was also observed (Tables 6, 7, 9).

At the beginning of the treatment phase, the enteral electrolyte solution HighAcetate promoted a gradual increase in blood pH, pCO_2_, HCO_3_^−^, BE, SID, Atot, and urinary pH (*p* < 0.05; Tables 5, 6, 10). The solution HighAcetate also promoted a reduction in the anion gap, correction of hyperchloremia over time (T_T_2h to T_T_12h), and a reduction in serum potassium and magnesium from T_T_4h onwards (*p* < 0.05; Tables 6–8).

In both treatments, during the treatment period (T_T_2h to T_T_12h), there was an increase in serum concentrations of ionic calcium and total calcium, blood glucose and urinary excretion of all electrolytes (Tables 4, 8, 9).

## Discussion

The intravenous administration of indSOL at a volume corresponding to 7.5% of body weight over 4 h induced moderate hyperchloremic metabolic acidosis in the animals. At the end of the induction phase, there was a reduction in blood pH, pCO_2_, HCO_3_^−^, base excess, SID, Atot, and urinary pH, along with an increase in the anion gap (Tables 3, 5, 6, 10). The efficacy of different protocols for the induction of hyperchloremic metabolic acidosis using 0.9% NaCl solution enriched with chloride was evaluated in adult horses, sheep, and neonatal calves, and all protocols were safe for inducing this disorder (17–20).

In the present study, the hyperchloremic metabolic acidosis induced in the mares was not comparable in intensity to the results observed by Romão et al., who induced a more intense hyperchloremic metabolic acidosis (Table 3). The volume of the acidifying solution was decreased by 25% and the infusion duration by 20% compared to their protocol (17), as our objective was to induce hyperchloremic metabolic acidosis of moderate intensity to minimally alter the animals’ well-being. All animals were subjected to the induction protocol twice, and the similar results observed in both treatments (HighAcetate and LowAcetate) during the induction phase (T_i_0h to T_i_4h) demonstrate the efficacy and safety of the induction protocol used in the present study.

The increase in blood sodium concentration during the induction phase (T_i_0h to T_i_4h) in both treatments was caused by indSOL, which contained a higher sodium concentration (154 mmol/L) than blood (Table 7). The effects of indSOL on natremia were reflected in urinary sodium excretion, which increased from T_i_2h onwards, reaching a peak at T_i_4h in both treatments (Table 9). The observed natriuresis is associated with the inhibition of sodium uptake by Na^+^-K^+^-2Cl^−^ cotransport in renal tubular cells due to increased blood and tubular chloride concentration, decreased tubular pH, and increased glomerular filtration rate (21, 22).

After 2 h of indSOL infusion (T_i_2h), there was a progressive increase in blood Cl^−^, reaching maximum values at T_i_4h (Table 7). Iatrogenic hyperchloremia was responsible for the increase in the anion gap and decrease in SID at T_i_2h and T_i_4h, which in turn caused a sharp decrease in pH, HCO_3_^−^, and base excess values. Furthermore, it triggered a compensatory response in the respiratory component by reducing pCO_2_ at the same assessment moments (23).

As observed with sodium, there was an increase in urinary chloride excretion starting at T_i_2h, peaking at T_i_4h (Table 9). These results are due to H^+^ elimination processes in the distal and collecting tubules, resulting in potassium reabsorption and the production of acidic urine, which is associated with hyperchloremia promoted by the infusion of indSOL (24). Similar results were reported by Romão et al. in studies on horses and sheep subjected to experimental hyperchloremic metabolic acidosis.

In both treatments, starting 2 h after the beginning of the induction phase (T_i_2h), an increase in blood potassium concentration was observed (Table 7), which persisted until 2 h after the start of treatment with enteral electrolyte solutions (T_T_2h). Despite this increase, the animals did not develop hyperkalemia, which differs from the results observed by Romão et al. (2017), who reported moderate hyperkalemia 5 h after starting an indSOL infusion in horses. The difference observed between the studies may be related to the shorter infusion time and volume of indSOL used in the present trial.

Although serum potassium remained within the reference range (25), the increase observed during the induction phase (T_i_0h to T_i_4h) can be attributed to physiological mechanisms of acid–base imbalance correction, specifically the exchange of K^+^ ions from intracellular fluid (ICF) with H^+^ ions from extracellular fluid (ECF) (25). This phenomenon occurs because inorganic acids are not evenly distributed between the ICF and ECF; in this case, the redistribution is only for the H^+^ ions. Intracellular buffering mechanisms act by neutralizing excess protons, generating a potential difference across membranes, which forces the displacement of intracellular cations to the ECF to maintain electroneutrality. As K^+^ is the main intracellular cation, its concentration in the blood increases as a result of this phenomenon (26, 27).

The reduction in urinary potassium excretion at T_i_2h was due to the renal control mechanism of acidosis (Table 9). Horses actively secrete H^+^ ions in the distal tubules and collecting ducts, allowing for the excretion of large quantities of protons and the reduction of the tubular fluid pH to minimum values close to 4.5. In this process, one HCO_3_^−^ and one K^+^ are reabsorbed for each H^+^ secreted, in addition to a chloride ion being passively secreted (28). During the induction phase (T_i_0h to T_i_4h), this increases in tubular reabsorption of potassium, combined with its translocation from the ICF to the ECF to buffer H^+^ ions, resulted in an increase in blood concentrations of this electrolyte. This culminated in greater glomerular potassium filtration and increased urinary excretion observed at the end of the induction phase (T_i_4h) and after 2 h of treatment (T_T_2h).

There was a slight increase in blood ionic calcium and an increase in urinary calcium in both treatments (Tables 8, 9). The binding of calcium to plasma proteins depends on pH; in acidemia, calcium is released from albumin to bind and buffer H^+^ ions. Furthermore, renal absorption of calcium is influenced by acid–base balance; in acute metabolic acidosis, calcium bound to the bone surface is released to buffer H^+^ ions, and tubular calcium reabsorption is inhibited through both transcellular and paracellular transport (29, 30). Reducing urinary pH in the tubular lumen increases the dissolution of calcium and magnesium in the urine, which results in increased excretion of these electrolytes as observed at T_i_2h and T_i_4h.

Considering that the animals were normohydrated at the beginning of the indSOL (T_i_0h) infusion, the volume of liquid administered intravenously was responsible for triggering hemodilution in the animals. In both treatments, hemodilution was responsible for the decrease total plasma protein (PPT) at T_i_2h and T_i_4h. The average decrease of 1 g/dL in PPT was reflected in Atot, resulting in a decrease in this variable at the respective times (Table 6). Cosenza et al. (31) and Pinto et al. (32) also observed a decrease in Atot in animals after intravenous hydration with electrolyte solutions at a rate of approximately 17 mL/kg/h and attributed it to the rapid volume expansion caused by the high rate of intravenous infusion of electrolyte solutions in normohydrated animals.

Volume expansion can be confirmed by the large increase in the volume of acidic urine observed in both treatments from T_i_2h, reaching the highest urinary volume (132 mL/min) and the lowest urinary pH (pH = 4.6) at T_i_4h (Table 10). In addition to the excess liquids administered to the animals being promptly eliminated in the urine, once the animals entered metabolic acidosis (T_i_2h) as observed in blood parameters, the kidneys began to act by excreting acidic urine (Table 10).

Increased production of acidic urine is one of the mechanisms the body uses to control the concentration of H^+^ ions in the extracellular fluid. The primary renal mechanisms for controlling acidosis promote the secretion of protons, the reabsorption of filtered HCO_3_^−^, and the synthesis of new HCO_3_^−^ (24). As mentioned previously, horses can actively secrete H^+^ ions, which allows for the elimination of large quantities of protons in the form of acidic urine (28).

Hyperchloremic metabolic acidosis was completely corrected by HighAcetate enteral electrolyte solution administered over 12 h at a rate of 15 mL/kg/h. After 2 h (T_T_2h), a difference between treatments began to occur. The administration of the HighAcetate solution generated lower BE values in the animals compared to the LowAcetate treatment (*p* < 0.05). This trend continued until the end of the treatment phase (Table 5). At T_T_12h, acidosis was corrected in animals that received the HighAcetate treatment, while those that received LowAcetate continued to have metabolic acidosis (Table 5). The same behavior was observed with blood pH, HCO_3_^−^, and DIF values at T_T_12h, as shown in Tables 5, 6.

These data suggest that enteral electrolyte solutions containing sodium acetate (EESAcNa), administered through a nasogastric tube in continuous flow, do not have immediate alkalinizing effects on the body when compared to the intravenous administration of sodium bicarbonate (33, 34). However, the time between the administration of enteral electrolyte solutions containing sodium acetate and their effect on the alkaline reserve can be considered short. As shown in Table 5, this effect begins after T_T_2h. The effectiveness and speed of the nasogastric route in horses in correcting electrolyte and acid–base imbalances were highlighted by Dias et al. (35) when comparing the effects of enteral electrolyte solutions and intravenous infusion of Ringer’s lactate.

The results indicate that EESAcNa can be used in patients with mild and moderate hyperchloremic metabolic acidosis. In patients with intense metabolic acidosis and very low BE values, the ideal approach is to administer sodium bicarbonate intravenously (33, 36). However, in some patients, a bolus of sodium bicarbonate can be administered intravenously, followed by EESAcNa administration. The advantage of EESAcNa is that, in addition to correcting electrolyte and acid–base imbalances, it also corrects dehydration, as it is infused in large volumes.

The correction of metabolic acidosis by the HighAcetate solution is due to the action of sodium acetate (NaAc) present in its composition. The effects of NaAc on acid–base balance have been described in the literature, and this substance is widely used as an alternative to sodium bicarbonate in enteral electrolyte solutions to hydrate calves with diarrhea (37, 38). In addition to its alkalizing effect, it also provides energy (39–41).

Acetate plays an essential role as an intermediate component in cellular oxidative metabolism. In the mitochondria, it undergoes conversion to acetyl-CoA, a process that consumes ATP. As acetyl-CoA, it participates in the tricarboxylic acid cycle, causing the consumption of three H^+^ ions during its metabolism into CO_2_ and H_2_O (42). These H^+^ ions are essential in the electron transport chain, where cytochrome oxidase, located in the inner membrane of the mitochondria, uses eight H^+^ molecules to produce H_2_O and ATP (11, 12). Consequently, the metabolism of an acetate molecule results in the consumption of 15 H^+^, contributing to buffering mechanisms in the body. This translates into an increase in blood PCO_2_ and HCO_3_^−^ concentration (41), confirming the results of the present study from T_T_2h.

Blood sodium concentrations remained constant in both treatments (Table 7). Although there was no significant difference in urinary sodium excretion between treatments, a slight reduction was detected during the treatment phase in comparison to T_i_4h (Table 9). Despite this decrease, urinary sodium excretion remained higher than the values observed before the beginning of the experimental period (T-12 h), as can be seen in Table 9. Although the HighAcetate and LowAcetate solutions contain less sodium than indSOL, the inhibition of indSOL induced renal sodium uptake persisted throughout the treatment phase. This inhibition was influenced by both the volume of liquids administered to the animals and the high concentrations of sodium, chloride, and potassium in the renal tubules (21, 22). It should be noted that these mechanisms are used by the kidneys to prevent the development of hypernatremia.

As for blood potassium, a gradual decrease was observed in both treatments, but the difference between them only appeared 6 h after the start of enteral fluid therapy (T_T_6h; Table 7). Urinary potassium excretion showed no difference between treatments, but progressively decreased over time, with a more pronounced decrease in animals that received the HighAcetate solution at T_T_10h (Table 9). This result was due to the action of the HighAcetate solution, which, when correcting hyperchloremic metabolic acidosis, caused the re-entry of potassium into the cells (43). This explains the decrease in blood and urinary concentrations of this electrolyte observed after the sixth hour of hydration (T_T_6h).

The treatments showed correction of hyperchloremia from T_T_2h onwards. The HighAcetate treatment showed lower chloride levels throughout the treatment period (T_T_2h - T_T_12h), with a significant difference between them emerging from T_T_12h onwards (Table 7). Serum chloride concentrations were also reflected in the reduction of its urinary excretion during the treatment phase (T_T_2h - T_T_8h) in both treatments, with a noticeable difference between treatments only at T_T_8h (Table 9). The reduction in blood chloride levels, combined with the increase in blood bicarbonate concentration, resulted in a decrease in the anion gap during the treatment phase in both cases (Table 6). These results can be attributed to the low concentration of chloride in both treatments (61 mmol/L), also contributing to the correction of hyperchloremic metabolic acidosis associated with sodium acetate metabolism (44).

When considering the strong ion theory, the correction of hyperchloremia appears as the main factor responsible for correcting blood pH and bicarbonate concentration (15, 23, 45). The enteral electrolyte solutions evaluated in this study showed a SID of 57.8 mmol/L and 12.9 mmol/L for HighAcetate and LowAcetate, respectively. Despite the similar chloride concentration in both treatments, the SID of the HighAcetate solution exceeded the plasma SID of adult horses (38–42 mmol/L). This caused a decrease in the concentration of chloride in the blood, generating an increase in plasma SID and a consequent alkalinizing effect (16). Therefore, the HighAcetate solution exerted a double alkalizing effect: firstly, by providing the body with an alkalizing substrate in the form of sodium acetate and, secondly, by having a higher SID than that of equine plasma.

Blood concentrations of total and ionic calcium increased in both treatments during the hydration phase (T_T_2h–T_T_12h; Table 8). In the LowAcetate treatment, the average ionic calcium values were slightly higher from T_T_6h onwards, with a significant difference appearing between treatments at T_T_12h. The expected increase in blood ionic calcium occurred because the concentration of this electrolyte in both treatments (11.35 mmol/L) exceeded the normal range for plasma calcium in horses (2.8–3.4 mmol/L) (46). Furthermore, in the LowAcetate treatment, the higher blood concentration of ionic calcium is associated with the prolonged maintenance of metabolic acidosis in the animals throughout the treatment phase. As mentioned previously, acidosis contributes to increased calcium mobilization from bones and its release from the fraction bound to plasma proteins (29, 47).

Correction of metabolic acidosis was expected to increase calcium absorption in the distal tubular lumen and subsequently correct or attenuate calciuria (30). Despite the expected effect, animals from both treatments showed an increase in urinary calcium excretion, possibly due to its high concentration in the composition of the electrolyte solutions used (Table 9). These results indicate that the amount of calcium present in the LowAcetate and HighAcetate treatments can be safely reduced without inducing hypocalcemia in the animals. Similar results were obtained by Monteiro et al. (2020).

In the HighAcetate treatment, serum magnesium decreased during the treatment phase, reaching its lowest value at T_T_10h. In contrast, in the LowAcetate treatment, the highest serum concentrations were observed throughout the treatment phase. Despite these variations, there was no significant difference between the treatments. The behavior of magnesium in the LowAcetate treatment is attributed to the maintenance of metabolic acidosis in the animals during this period. This observation aligns with the findings of Blumberg et al. (48), where acute metabolic acidosis led to hypermagnesemia by inducing the release of magnesium bound to plasma proteins. This mechanism, similar to the previously mentioned protein-bound calcium, serves as a buffering strategy for free H^+^ by plasma proteins.

Regarding urinary magnesium excretion, the increase observed in the induction phase was maintained during the treatment phase in both treatments, with no significant difference between them (Table 9). These results differ from those observed by Ribeiro Filho et al. (49). However, the present trial demonstrates that the presence of 0.3 g/L of magnesium chloride in both enteral electrolyte solutions provided the animals with an adequate amount to avoid hypomagnesemia due to hemodilution.

The glycemic rate of animals in both treatments increased during the period in which they received enteral electrolyte solutions (Table 4). As found by Ribeiro Filho et al. (49), the use of dextrose and maltodextrin in amounts less than 15 g/L in enteral electrolyte solutions is a good source of energy for sick horses without triggering lactic fermentation in the animals’ large intestine. In the present study, this is confirmed by plasma lactate concentrations. In both treatments, there was a small increase in lactate concentrations during the treatment phase (T_T_2h—T_T_12h; Table 4). However, this increase was not clinically relevant, as the values remained within the reference range of 1.1 to 1.78 mmol/L (46).

The HighAcetate treatment presented the lowest mean urine flow values, differing from the LowAcetate treatment at T_T_4h, T_T_8h, and T_T_12h (Table 10). Despite this decrease, the volume of urine produced was still about 10 times the average volume of urine observed before the start of the experimental phase (T-12 h). The greater volume of urine produced in the LowAcetate treatment is associated with the difference of almost 100 mOsm/L between the LowAcetate (196 mOsm/L) and HighAcetate (290 mOsm/L) treatments. As demonstrated by Monteiro et al. (2020), hypotonic enteral electrolyte solutions promote greater volume expansion, triggering a higher glomerular filtration rate, and therefore, greater urine production.

Urinary pH also reflected the changes in the animals’ acid–base balance during the treatment phase. In the HighAcetate treatment, there was a gradual increase in urinary pH that reached the highest value at T_T_12h, differing from the LowAcetate at T_T_10h (Table 10). These results confirm what was observed in relation to blood markers of acid–base balance: at the end of treatment with the HighAcetate electrolyte solution (T_T_12h), hyperchloremic metabolic acidosis had already been corrected in the animals receiving this treatment, consequently ending the aciduria. In contrast, in the LowAcetate group, aciduria persisted, as indicated in Table 10, with hyperchloremic metabolic acidosis continuing until the end of the experimental phase (T36h).

The present study demonstrated the efficacy and safety of the HighAcetate enteral electrolyte solution, containing 8 grams of sodium acetate per liter of solution, administered at a rate of 15 mL/kg/h for 12 h in continuous flow, in the correction of hyperchloremic metabolic acidosis in adult horses. These results contribute significantly to the equine medicine, providing a new generation of alkalizing enteral electrolyte solutions for the treatment of hyperchloremic metabolic acidosis in horses without the adverse effects of sodium bicarbonate when used as a component of enteral electrolyte solutions. Additional investigations involving sick animals are imperative to confirm the clinical efficacy of this alkalizing enteral electrolyte solution.

## Data Availability

The original contributions presented in the study are included in the article/supplementary material, further inquiries can be directed to the corresponding author.
